# Proteasome stress sensitizes malignant pleural mesothelioma cells to bortezomib-induced apoptosis

**DOI:** 10.1038/s41598-017-17977-9

**Published:** 2017-12-15

**Authors:** Fulvia Cerruti, Genny Jocollè, Chiara Salio, Laura Oliva, Luca Paglietti, Beatrice Alessandria, Silvia Mioletti, Giovanni Donati, Gianmauro Numico, Simone Cenci, Paolo Cascio

**Affiliations:** 1 Department of Veterinary Sciences, University of Turin, Largo P. Braccini 2, 10095 Grugliasco, Turin Italy; 2 Medical Oncology Unit, Ospedale U. Parini, Viale Ginevra 3, 11100 Aosta, Italy; 3 San Raffaele Scientific Institute, Division of Genetics and Cell Biology, Via Olgettina 60, 20132 Milan, Italy; 4 Thoracic Surgery Unit, Ospedale U. Parini, Viale Ginevra 3, 11100 Aosta, Italy; 5 Medical Oncology, Azienda Ospedaliera SS Antonio e Biagio e C Arrigo, Via Venezia 16, 15121 Alessandria, Italy

## Abstract

Based on promising results in preclinical models, clinical trials have been performed to evaluate the efficacy of the first-in-class proteasome inhibitor bortezomib towards malignant pleural mesothelioma (MPM), an aggressive cancer arising from the mesothelium of the serous cavities following exposure to asbestos. Unexpectedly, only minimal therapeutic benefits were observed, thus implicating that MPM harbors inherent resistance mechanisms. Identifying the molecular bases of this primary resistance is crucial to develop novel pharmacologic strategies aimed at increasing the vulnerability of MPM to bortezomib. Therefore, we assessed a panel of four human MPM lines with different sensitivity to bortezomib, for functional proteasome activity and levels of free and polymerized ubiquitin. We found that highly sensitive MPM lines display lower proteasome activity than more bortezomib-resistant clones, suggesting that reduced proteasomal capacity might contribute to the intrinsic susceptibility of mesothelioma cells to proteasome inhibitors-induced apoptosis. Moreover, MPM equipped with fewer active proteasomes accumulated polyubiquitinated proteins, at the expense of free ubiquitin, a condition known as proteasome stress, which lowers the cellular apoptotic threshold and sensitizes mesothelioma cells to bortezomib-induced toxicity as shown herein. Taken together, our data suggest that an unfavorable load-versus-capacity balance represents a critical determinant of primary apoptotic sensitivity to bortezomib in MPM.

## Introduction

The 26S proteasome is an ATP-dependent protease complex abundantly expressed in eukaryotic cells, responsible for the regulated hydrolysis of most cellular proteins^1^. The 26S is a multi-subunit enzyme consisting of a 20S particle, where polypeptides get hydrolyzed, associated with the 19S regulatory particles, responsible for recognizing, unfolding, and translocating polyubiquitinated proteins into the inner degradative chamber of the 20S^2^. This is a ~700 kDa cylindrical macromolecular machine formed by four overlapping heptameric rings, consisting of α (the outer rings) or β (the inner rings) subunits^3^. Normally, the hydrolyzing activity is yielded by subunits β5, β2, and β1 of the 20S proteasome. However, cells of the immune system or exposed to proinflammatory cytokines are induced to express three additional homologous subunits (β5i, β2i, β1i), which replace their constitutive counterparts in newly synthesized and shorter-lived immunoproteasomes^4^. In principle, proteasomes can hydrolyze the C-terminal amide bond of every amino acid but proline^5^; however, proteolytic activities assessed by means of short fluorogenic peptides identify three defined hydrolyzing preferences: trypsin-like (i.e. hydrolysis at the C-terminus of basic residues, β2/β2i), chymotrypsin-like (i.e. hydrolysis at the C-terminus of hydrophobic residues, β5/β5i), and caspase-like activity (i.e. at the C-terminus of acidic residues, β1/β1i). A variety of low molecular weight inhibitors of the proteolytic sites of 20S have been identified that can enter cells and block proteasomal protein degradation^6^. These agents proved essential for investigating the biological role of the ubiquitin-proteasome system and led to the discovery of diverse key regulatory functions of this pathway. Furthermore, since proteasome inhibitors (PIs) also induce adaptive and maladaptive responses (e.g. the unfolded protein and heat-shock responses), showing previously unpredicted specificity against certain tumor cells, they became the paradigm of negative proteostasis regulators in cancer therapy^7^.

The anti-cancer use of PIs stemmed from the original observation of their remarkable toxicity against a variety of cancer cells at doses that had little or no toxicity against normal, non-transformed cells^8^. Further studies and clinical trials allowed the rapid approval of the modified boronic dipeptide bortezomib (Btz, PS-341 or Velcade®) for the treatment of multiple myeloma (MM)^9^ and refractory mantle cell lymphoma^10^. Encouraging results were also reported on other hematological and solid cancers^11,12^. Moreover, the anti-tumor activity of five second generation PIs is currently being evaluated in dedicated clinical trials^13^. In the case of MM, the accumulation of polyubiquitinated proteins is an established mechanism of PI-induced apoptosis^14,15^. Such a condition, referred to as *proteasome stress* ^16,17^, in plasma cells is particularly aggravated by the extremely high load of protein degradation imposed by large-scale immunoglobulin production, which makes the apoptotic threshold particularly low^18–20^. This *load-versus-capacity* model offers an explanation for the exquisite sensitivity of malignant plasma cells to PIs^21^, and also for the variability observed among different MM cell lines and primary tumors, which differ in both proteasome capacity and degradative workload^18^. Furthermore, demonstrating cause-effect relationships, increasing proteasome expression^18^ and reducing protein synthesis^21^ independently increased bortezomib resistance. Whether and to what extent the load-versus-capacity model may also contribute to explain PI responsiveness of other cancers, and particularly solid tumors, is currently unknown.

Malignant pleural mesothelioma (MPM) is a highly deadly cancer arising from the mesothelium of serous cavities following exposure to asbestos^22^. Circa 3,000 new MPM cases are diagnosed yearly in the United States, with ~250,000 deaths predicted to be caused by MPM in the next 30 years in occidental Europe^23,24^. Although measures have been implemented to limit further exposure to asbestos, the long latency of MPM generated a strikingly increasing incidence of this malignancy. MPM is normally intractable by to local therapies and typically progresses with a median overall survival of 12–36 months for localized illness and only 8–14 months for advanced disease^25^. The currently available chemotherapeutic drugs are poorly active against MPM, with usual single-agent response rates of ≤20%^26^, and first-line radiation therapy is generally ineffective^27^. Combination therapy with pemetrexed and cisplatin is the current standard first-line treatment^28^. However, median survival on this regimen is below 1 year, with <50% response rate. Notable, a randomized phase 3 trial recently reported a significant gain in overall survival (median 18.8 *vs*. 16.1 months) in MPM patients treated with bevacizumab in addition to the standard pemetrexed and cisplatin regimen^29^. Moreover, no approved standard of care for patients who have relapsed is available, since several trials have failed to show useful activities in a second-line setting^30^. Hence, identifying and developing new treatments for MPM is an unmet need.

Although some molecular profiling studies of malignant mesothelioma identified the ubiquitin-proteasome system (UPS) as a potential therapeutic target^31–33^, several others could not establish any significant correlation between clinical and/or anatomo-histological features of MPM and alterations of the proteasome proteolytic pathway^34–41^. Notwithstanding, the first-in-class PI bortezomib demonstrated promising anti-tumoral activity in both *in vitro* and *in vivo* preclinical models of MPM. Specifically, bortezomib was shown to induce cytotoxicity, cell cycle arrest, and apoptosis in several primary patient-derived and immortalized MPM cell lines, while sparing normal mesothelial cells^42–44^. The effector mechanisms of such toxicity are poorly understood, with an intricate network of pro- and anti-apoptotic factors possibly implicated^45–48^. These results provide the rationale to clinically evaluate the effect of bortezomib, alone or in combination with cisplatin, against MPM^49,50^. However, only modest therapeutic benefits were observed. In particular, data from a multicenter Phase II study of bortezomib as monotherapy in an unselected population of MPM patients demonstrated a poor (5%) response rate, implying that inherent mechanisms of resistance protect primary tumors^49^. Identifying the molecular bases of the differential sensitivity of MPM cells to proteasome inhibition is, therefore, critical to design novel pharmacologic strategies aimed at increasing responsiveness to bortezomib, alone or in therapeutic combinations. To this end, we hereby challenged the load-versus-capacity model in a panel of four human MPM cell lines characterized by differential apoptotic sensitivity to bortezomib.

## Results

### Human MPM cell lines show differential sensitivity to the pro-apoptotic effects of bortezomib

**Figure 1:**
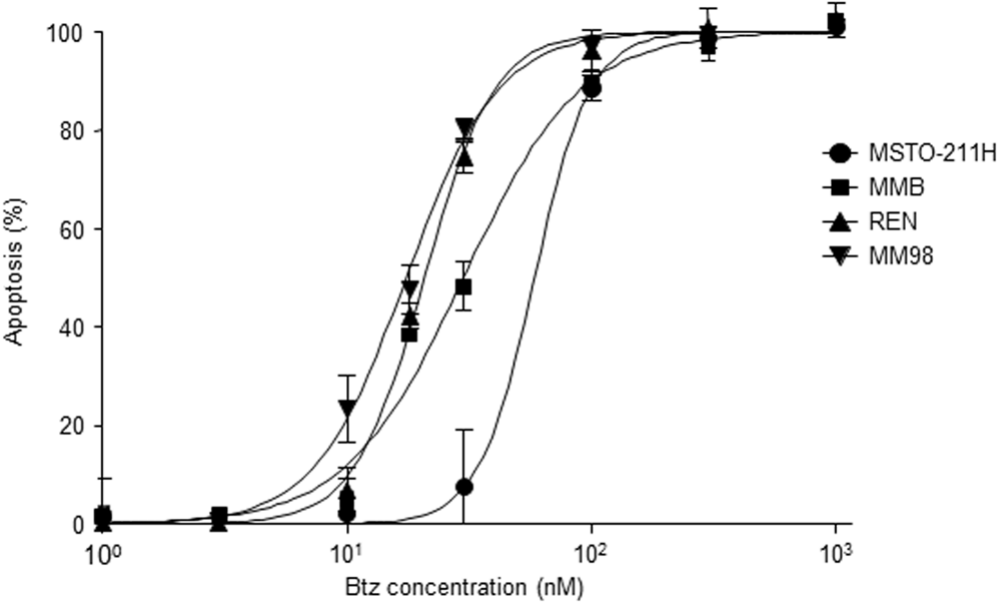
Effects of bortezomib on the viability of malignant pleural mesothelioma cell lines. MM98, REN, MMB, and MSTO-211H cells were treated with increasing concentrations of bortezomib and apoptosis was measured after 48 hours by FACS of annexin V and propidium iodide positive cells, and expressed as variation vs. untreated cells. Values are mean ± SE of three independent experiments. R^2^ for a sigmoidal dose-response curve ≥0.9 in all cases.

In an effort aimed at verifying whether different MPM cell lines display differential sensitivity to apoptosis triggered by proteasome inhibition, we initially tested the biological effects of bortezomib on a panel of four human MPM clones. In particular, MSTO-211H, REN, MM98, and MMB cell lines were treated with increasing amounts of bortezomib and apoptosis was measured after 48 hours by FACS assessment of annexin V and propidium iodide positive cells. As shown in Fig. 1, all clones displayed a marked dose-dependent susceptibility to the pro-apoptotic effects of bortezomib in the nanomolar range. However, the sensitivity to the inhibitor of the four cell lines clearly differed. In particular, MM98 and REN cells displayed EC_50_ values for bortezomib-induced apoptosis of 17 and 22 nM respectively, while the MSTO-211H line was relatively more resistant with an EC_50_ of 60 nM. The MMB line was characterized by an intermediate sensitivity (EC_50_ 33 nM; Table 1). Thus, we demonstrated that the aforementioned MPM lines are characterized by a well-defined differential sensitivity/resistance to bortezomib and are therefore suitable for investigating the molecular mechanisms responsible for the different susceptibility of MPM cells to apoptosis caused by proteasome inhibition.

**Table 1:** Differential sensitivity of MPM cell lines to bortezomib.

Cell line	EC_50_ Btz (nM)
MM98	16.9 ± 1.7
REN	22.1 ± 1.4
MMB	33.4 ± 2.0
MSTO-211H	60.1 ± 8.6

The Malignant Pleural Mesothelioma cell lines MM98, REN, MMB, MSTO-211H were treated for 48 hours with increasing concentrations of bortezomib (Btz) and the proportion of apoptotic (annexin V + propidium iodide-) cells was measured by FACS. The indicated EC_50_ are an average of 3 independent experiments ± SE.

### Different sensitivity of MPM clones to bortezomib correlates with proteasome activity

**Figure 2:**
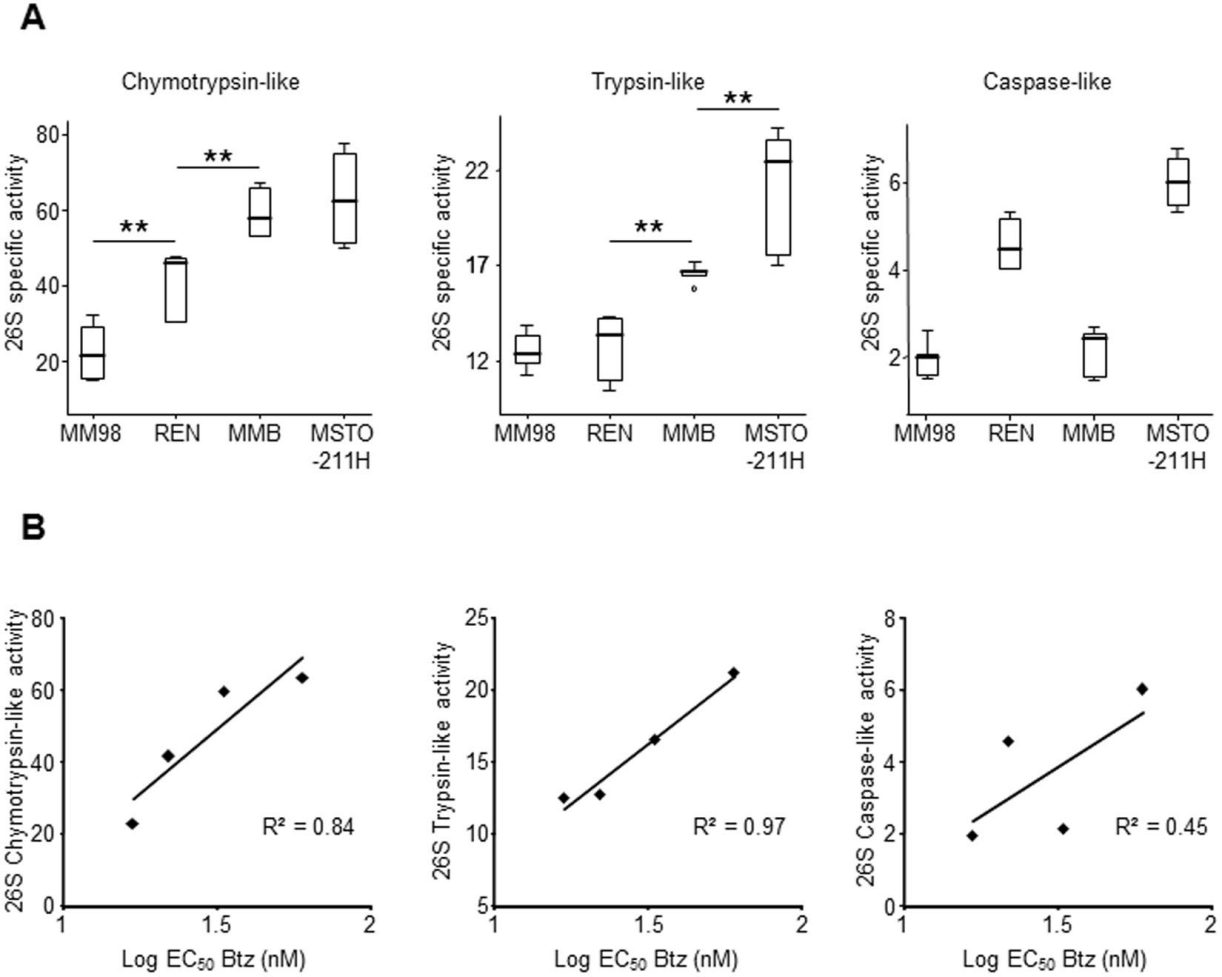
Reduced proteasome activity in MPM cell lines with increased sensitivity to bortezomib. (**A**) Chymotrypsin-like, trypsin-like, and caspase-like specific activities of proteasomes were assessed in cell extracts as described in the Materials and Methods and expressed on a per-cell basis. Data are the mean of six independent measurements. **P < 0.01. (**B**) Correlations of 26S proteasome peptidase activities and EC_50_ values relative to the apoptotic effect of bortezomib in MPM cell lines.

To assess whether in MPM, as in the paradigmatic PIs-sensitive cancer MM, enhanced susceptibility towards cytotoxic effects of bortezomib correlates with lower overall potential proteasomal proteolytic capacity, we measured the three main peptidase activities of 26S proteasomes in cellular extracts from the four MPM clones by specific fluorogenic substrates in the presence of ATP. With this method proteasome cleavage specificities can be selectively assessed in crude extracts, avoiding time-consuming and laborious chromatographic purifications, since the contribution of non-proteasomal enzymes is determined by highly specific inhibitors of proteasome active sites^51^. By this approach, we could demonstrate that rates of fluorogenic peptides hydrolysis clearly differ between the MPM lines (Fig. 2A). More importantly, the strongly Btz-sensitive and relatively BTZ-resistant MM98 and MSTO-211H lines displayed, respectively, lower and higher 26S chymotrypsin-like and trypsin-like activities, which are the main proteasomal cleavage specificities and the rate limiting activities for protein turnover^52,53^. Moreover, for all MPM clones a clear, direct correlation between proteasome chymotrypsin- and trypsin-like activities and resistance to apoptosis induced by bortezomib was seen (Fig. 2B), strongly suggesting that the size of the proteasomal compartment contributes to the sensitivity to PIs in mesothelioma cells. Only for the minor caspase-like activity could a clear positive correlation with Btz-EC50 values of the corresponding MPM lines not be established (Fig. 2B).

### Loss of concerted expression of proteasomal subunits in MPM clones

**Figure 3:**
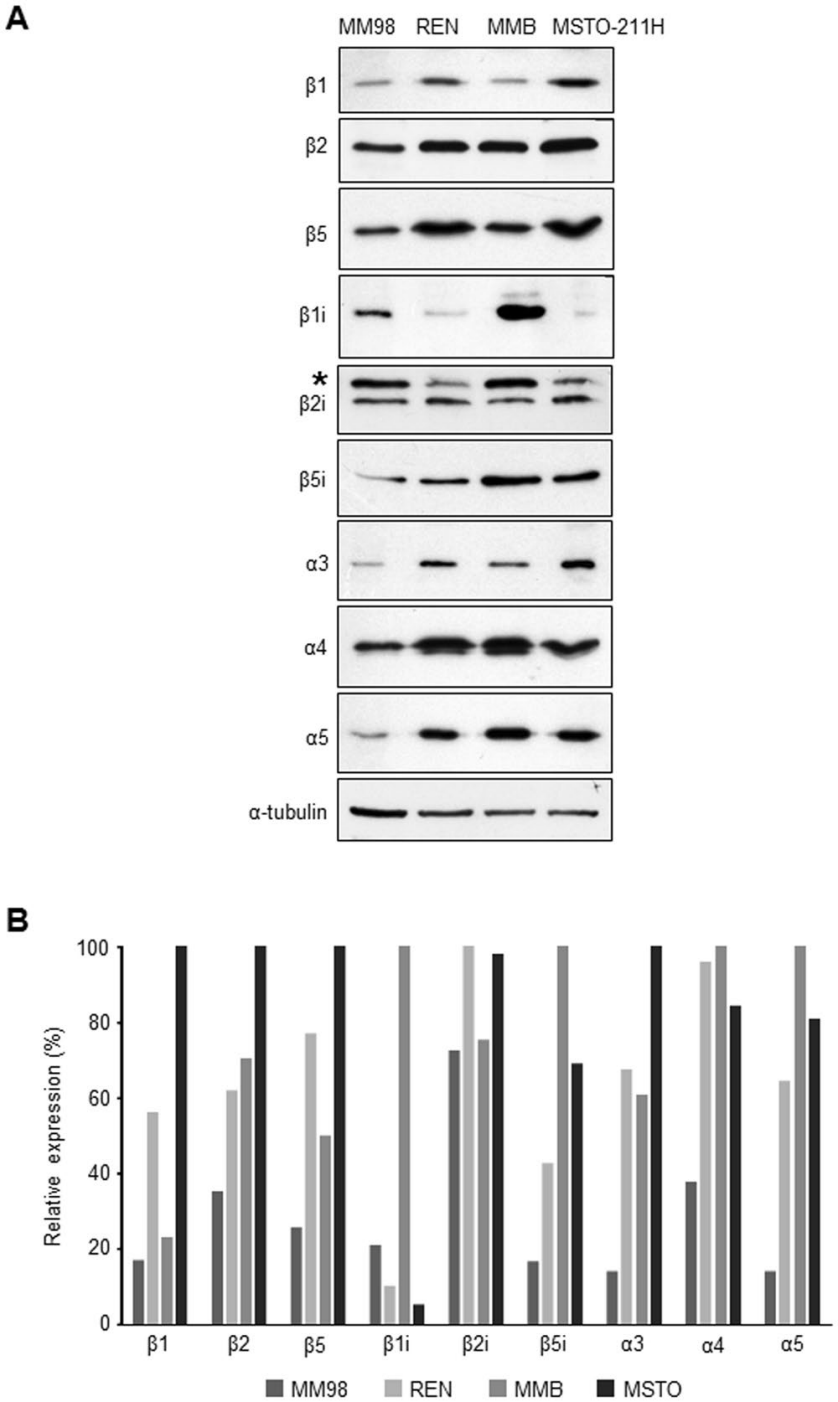
Steady-state levels of catalytic β and non-catalytic α-subunits of constitutive and immunoproteasomes in MPM cell lines. (**A**) Cellular extracts from the MM98, REN, MMB, and MSTO-211H lines were resolved by SDS-PAGE and analyzed by western blotting with Abs to different proteasome catalytic β and non-catalytic α-subunits. Equal protein amounts (6 µg) were loaded in each lane, with α-tubulin serving as a loading control. *Unspecific band. All the blots were cropped according to their locations in the membrane in order to fit the size, see the original figures in the supplementary information. (**B**) Relative densitometric quantification of western blot bands shown in panel A normalized by the control loading signal.

To investigate the reasons for the difference in proteasome activities of MPM clones in more detail at the molecular level, we measured the expression levels of several proteasome subunits by western blot analysis. In this regard, it is worth noting that the steady-state levels of mature β-subunits, which are subjected to autocatalytic cleavage upon assembly to generate the active form, are directly responsible for proteasome peptidase activities^54^. Unexpectedly, the expression patterns of the catalytic β-subunits of both constitutive and immuno proteasomes was complex and highly variable between MPM lines (Fig. 3A). In fact, indicative of a loss of the regulatory mechanisms that normally ensure concerted expression of the catalytic subunits of constitutive and immuno proteasomes, each clone showed an individual pattern of content of β-subunits, with some antigens clearly up-regulated and others down-regulated, with no clear delineations between the different lines (Fig. 3B). Similarly, the steady-state levels of the α-subunits also appeared variable and not concertedly regulated in each MPM line, although in this case expression levels seemed concordantly reduced in MM98 cells, which are characterized by lower proteasome activity (Fig. 2). However, α-subunits do not necessarily reflect assembled, functional proteasomes, since they also include the pool of free intracellular subunits^54^.

### More Btz-sensitive MPM lines display higher levels of proteasome stress

**Figure 4:**
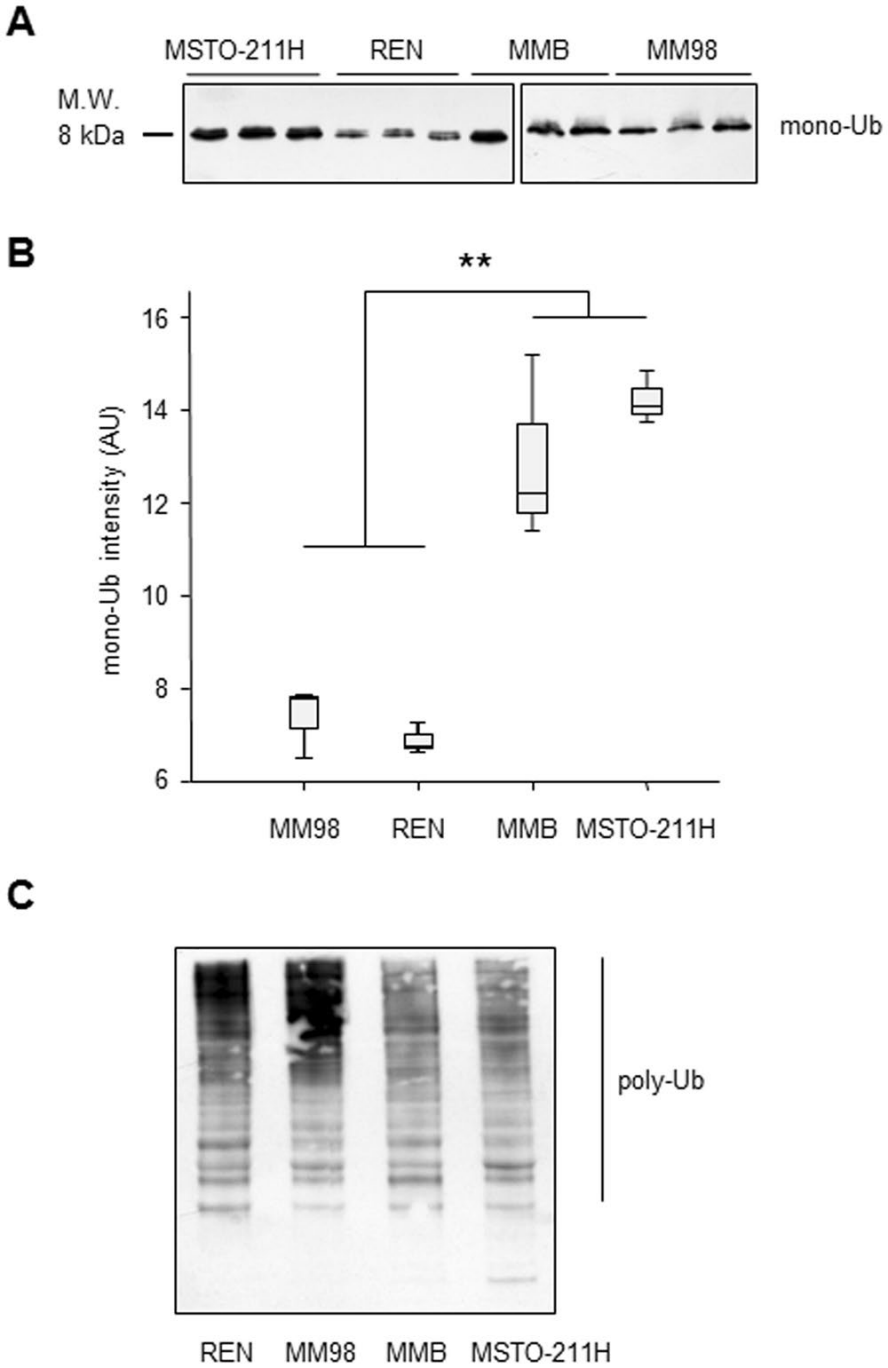
Basal accumulation of polyubiquitinated proteins at the expense of the pool of free ubiquitin in MPM cell lines more sensitive to bortezomib. (**A**) 15 µg of cellular extracts from the MM98, REN, MMB, and MSTO-211H lines were incubated with anti-ubiquitin (Ub) Ab. For each clone, data from three independent cell extracts are shown. Samples derive from the same experiment and that gels/blots were processed in parallel. The blots were cropped according to their locations in the membrane in order to fit the size, see the original figures in the supplementary information. (**B**) Densitometric quantification of mono-ubiquitin bands shown in panel A. **P < 0.01. (**C**) Polyubiquitinated proteins (poly-Ub, 5 µg of cellular extract) were resolved as a smear in an 12% SDS-Page gel, while free Ub was separated in an 18% gel.

A reduced proteasomal compartment may not be effectively suited to cope with the degradative needs of the cell. As a consequence of the resulting unbalance in the load-versus-capacity ratio, cells are expected to experience a higher basal level of proteotoxic stress that, in turn, lowers their intrinsic apoptotic threshold. To verify this scenario, we therefore looked for signs of proteasomal sufferance in the MPM lines by assessing the intracellular levels of free and polymerized ubiquitin. As shown in Fig. 4A, western blotting analysis unambiguously demonstrated that the more Btz-sensitive MM98 and REN clones are characterized by a significantly lower content of unbound ubiquitin compared to the relatively more resistant MMB and MSTO-211H cell lines (Fig. 4B). This deficiency of free ubiquitin reflects its role in building polyubiquitin chains, which, in fact, were greatly accumulated in the more Btz-sensitive clones (Fig. 4C).

**Figure 5:**
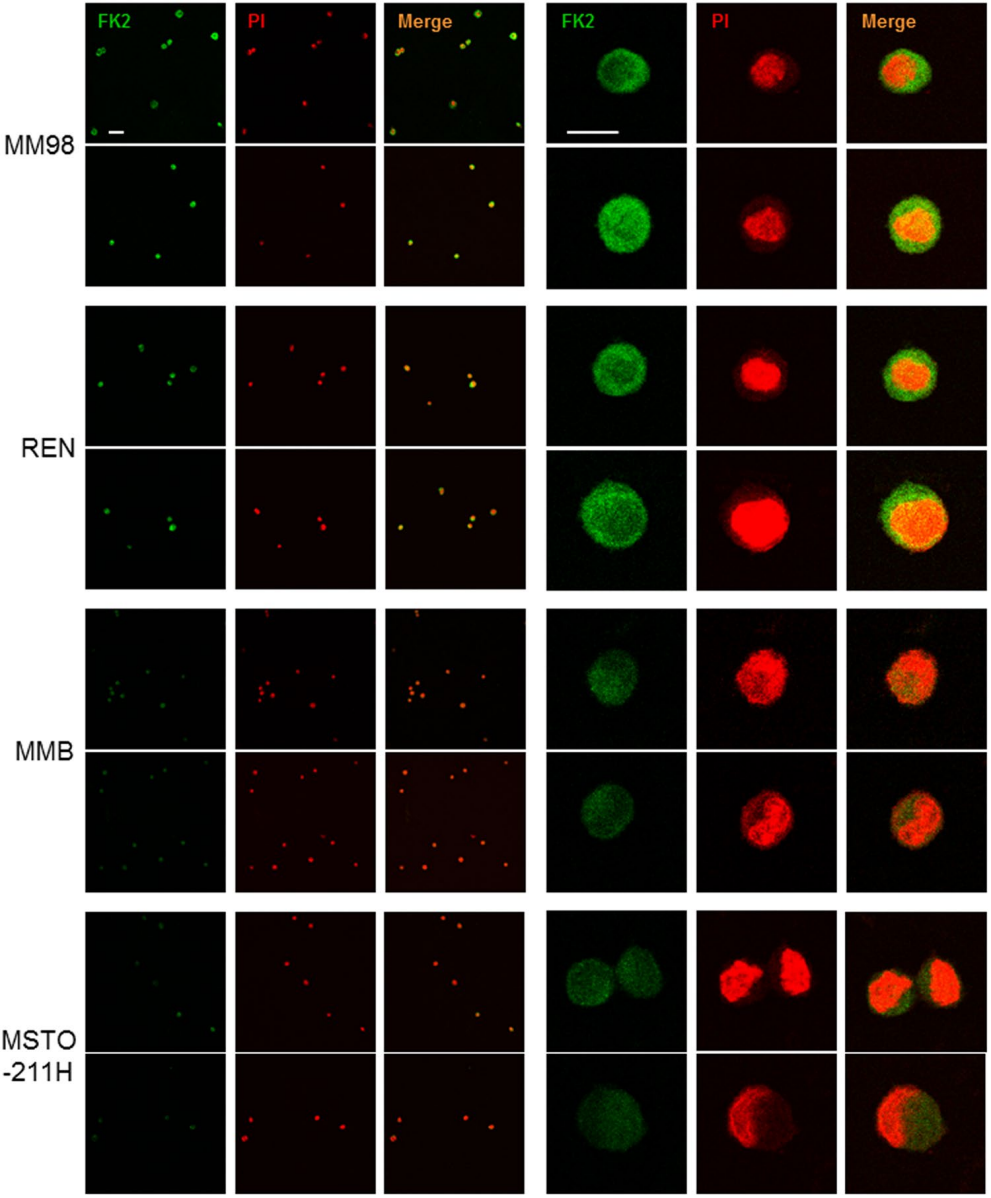
Immunofluorescence microscopy of polyubiquitinated proteins in MPM cell lines. Cells were stained with FK2 antibody, which specifically recognizes poly-ubiquitin chains, and analyzed by confocal microscopy in three independent experiments. Nuclei labelled red by propidium iodide (PI); size bar: 50 μm lower magnification (left) and 10 μm higher magnification (right). Representative images. Cell area (µ^2^), mean ± St. dev.: MM98 108 ± 11, REN 117 ± 9, MMB 93 ± 12, MSTO-211H 110 ± 8.

**Figure 6:**
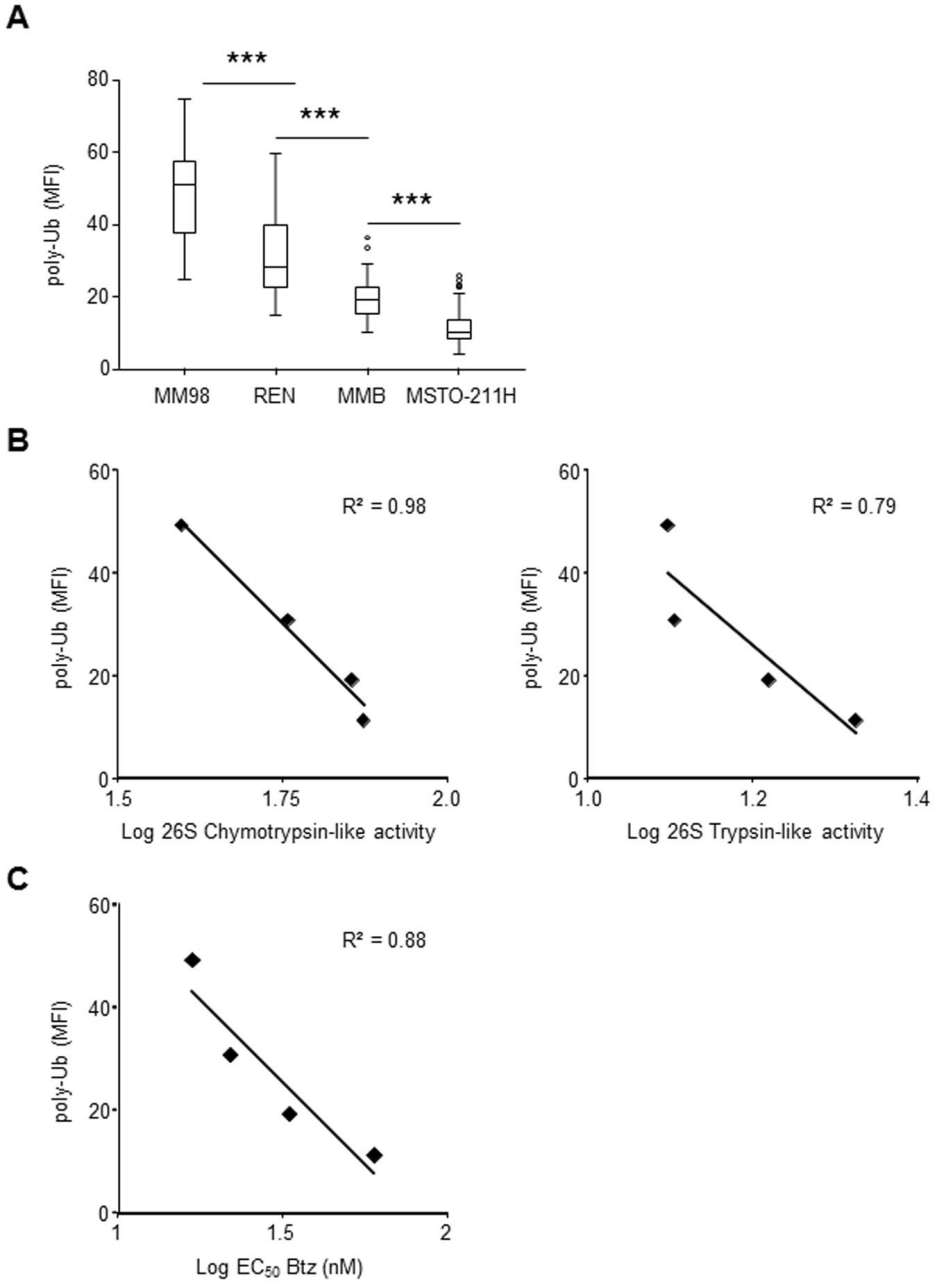
Basal accumulation of polyubiquitinated proteins inversely correlates with proteasome activity and with intrinsic resistance of MPM clones to the pro-apoptotic effects of bortezomib. (**A**) Different levels of polyubiquitin conjugates in MPM cell lines. Quantification of the FK2-dependent fluorescence in MPM cells was performed using Image J software (n ≥ 100 cells in ≥ 16 distinct fields). ***P < 0.001. Inverse correlation between levels of polyubiquitinated proteins in MPM clones and 26S proteasome chymotrypsin- and trypsin-like activities (**B**) and EC_50_ values relative to apoptotic sensitivity to bortezomib (**C**).

To investigate these findings, indicating saturation of proteasome proteolytic capacity with consequent accumulation of undegraded substrates in MPM cells that were more vulnerable to bortezomib, we evaluated polyubiquitinated proteins at the single cell level by confocal immunofluorescence microscopy. To this aim, we used FK2, an antibody that recognizes different types of polyubiquitin chains^55^ and therefore it is particularly well-suited to detect all potential proteasome substrates. In fact, several recent evidences indicate that not only the canonical lysine-48 linked polyubiquitin chains but also other alternative ubiquitin linked chains support degradation by the 26S proteasome^56,57^. In line with the western blot data, this analysis revealed strong basal accumulation of polyubiquitinated proteins in REN and MM98 cells with a discrete cytosolic and nuclear pattern, clearly exceeding the signal present in MMB and MSTO-211H cells (Fig. 5). Subsequent quantification and statistical analysis of FK2-dependent fluorescence confirmed that basal accumulation of polyubiquitinated proteins differed substantially in all four MPM clones (Fig. 6A). Most importantly, the fluorescence intensity associated to polyubiquitinated proteins negatively correlated both with the main functional activities of 26S (Fig. 6B) and the EC_50_ of the corresponding MPM clone for the pro-apoptotic effect of bortezomib (Fig. 6C), clearly indicating a role for overload of proteasomal degradative capacity in determining cellular susceptibility to PIs.

## Discussion

Previous studies have shown that bortezomib and other PIs exhibit significant anti-tumoral activity in preclinical models of MPM both *in vitro* and *in vivo* ^42–44,58,59^. Disappointingly, however, this efficacy was not successfully translated in clinical activity due to either primary and/or acquired resistance^49,50^. In an effort aimed at identifying the mechanisms of the different intrinsic sensitivities of MPM cells to cytotoxicity induced by proteasomal inhibition, we initially assessed the pro-apoptotic effects of bortezomib in a panel of four human MPM cell lines. In accordance with published data, this analysis revealed that all four MPM clones tested displayed pronounced apoptotic vulnerability to bortezomib, with EC_50_ values comparable to those determined by the same experimental approach for different cell lines of human multiple myeloma, the paradigmatic proteasome inhibitors responsive cancer^21^. Crucially, however, the primary sensitivity of these MPM lines to bortezomib varied, with MM98 and REN cells characterized by higher vulnerability to the drug (EC_50_ values of 17 and 22 nM, respectively), while the MSTO-211H line was relatively more resistant (EC_50_ of 60 nM) and the MMB clone hallmarked by intermediate susceptibility (EC_50_ of 33 nM). The finding that these mesothelioma cell lines are not equally responsive to the cytotoxic effects of bortezomib prompted us to investigate the molecular mechanisms of their different intrinsic sensitivity to PIs. In fact, primary resistance to bortezomib represents a crucial therapeutic challenge not only for solid tumors like MPM, but also for prototypical PIs-responsive hematological malignancies. More than 50% of MM patients, for example, fail to respond to bortezomib and the basis of this different intrinsic responsiveness remains elusive. We^18,21^ and others^60^ have recently shown that exquisite apoptotic sensitivity of both primary or immortalized MM cells arises from exuberant synthesis of abnormal proteins impinging on a reduced proteasome pool. The resulting saturation of cellular proteolytic capacity (a condition referred to as *proteostenosis*) has profound consequences for cell viability and tumor chemosensitivity^21^.

The present study is, to the best of our knowledge, the first documenting that different levels of basal proteasome stress, intended as a situation of precarious equilibrium, characterized by lack of overt signs of cell sufferance (e.g. cell cycle arrest), but deeply affecting the capacity of the cell to successfully cope with additional stress, underlies the intrinsic apoptotic sensitivity to bortezomib for a solid cancer such as pleural mesothelioma. Under normal conditions mammalian cells are capable of adapting their proteasome content to proteolytic needs (through complex feedback loops involving the transcription factor NRF1, and the mTorc1 and ERK5 pathways), thus relieving proteasome stress, as demonstrated in several experimental settings^61,62^. However, as shown in many studies, cancer cells generally suffer from more stress than their benign counterparts, and are generally more reliant on stress adaptive pathways, making them less efficient at coping with additional stress, hence the model of non-oncogene addiction, which provides a rationale for targeting protein homeostasis or other stress adaptive pathways^63^. Consistent with this concept, the neoplastic cell lines (both in the case of MPM or MM) hallmarked by an intrinsic, exquisite sensitivity to PI-induced cytotoxic effects, invariably show pathognomonic signs of proteasome stress (e.g. marked accumulation of polyubiquitinated conjugates at the expense of free monomeric ubiquitin), even in the absence of any other condition or stimulus that might negatively impinge on proteasome cellular content or activity. Although other mechanisms such as differential activities of ubiquitin-ligases or deubiquitinating enzymes might contribute to the increase in steady-state levels of ubiquitin-conjugates, the strong correlation between accumulation of polyubiquitinated substrates and low chymotrypsin-like and trypsin-like proteasome activities (that are rate limiting for degradation of proteins *in vivo* and *in vitro* ^52,53^) strongly indicates that reduced proteasome levels and activities play a pivotal role in this process. In fact, in MPM, as already well documented for MM, enhanced levels of proteotoxic stress invariably correspond to a strong reduction of the cellular proteasome compartment. Indeed, the two MPM lines (MM98 and REN) displaying the lower overall 26S activity were found to accumulate higher levels of polyubiquitinated proteins than the two lines (MMB and MSTO-211H) characterized by a higher proteasome content. Interestingly, our results show that proteasome levels may vary greatly among different MPMs, with profound implications for the intrinsic capability of coping with cytotoxic stress, given the key role of the proteasome in integrating signals that control cell-cycle progression, apoptosis, and metabolism. Accordingly, chymotrypsin-like and trypsin-like proteasome activities inversely correlate with the sensitivity to bortezomib of MPM lines, indicating that reduction of the proteasome cellular pool represents a crucial determinant of proteotoxic stress that predisposes mesothelioma cells to the pro-apoptotic effects of PIs. Importantly, bortezomib mainly targets chymotrypsin-like proteasomal activity, while discordant results were reported concerning its effects toward trypsin-like sites^11,64^. However, it is worth noting that *in vivo* both bortezomib and other PIs exert their toxic effects by blocking catabolism of full length proteins, whose rates of hydrolysis are determined by the concomitant and coordinated action of all three proteasome active sites, acting through a complex (and not yet completely understood) network of allosteric functional interactions^65,66^.

Moreover, western blot analysis of the steady-state levels of several proteasome subunits revealed that the MPM cells analyzed in our study have lost the standard, concerted expression of all three catalytic β-subunits of constitutive or immuno proteasomes. On the contrary, each MPM clone shows a specific and individual pattern of catalytic β-subunit expression, indicative of a loss of the regulatory mechanisms that under physiological conditions ensure coordinated expression of active β-subunits, with the purpose to adapt proteasome capacity to cellular needs so as to maintain homeostasis^61^. Such dysregulated expression patterns may be a consequence of genotoxic stress typical of neoplastic cells, and are likely to account for the difference in the proteasome activity between MPM cell lines that our study unveiled. In fact, reduced expression of individual catalytic subunits and conceivable formation of non-standard mixed proteasomes containing β-subunits of both immuno and constitutive proteasomes may substantially affect overall proteasome enzymatic efficiency^67^. Of note, loss of the concerted, physiological expression of all proteasome subunits is likely to account for the inability of several DNA microarrays studies to demonstrate a crucial role of the proteasome proteolytic pathway in the biology and pathogenesis of MPM^34–41^. These results emphasize the need to integrate microarrays expression data with functional measurements of proteasome enzymatic activities.

Of great interest, our study revealed a striking positive correlation between high levels of polyubiquitinated conjugates in MPM cell lines and primary sensitivity to the pro-apoptotic effects of bortezomib, which strongly suggests a cause-effect relationship. In fact, the more Btz-sensitive MM98 and REN lines displayed high levels of basal proteasomal stress indicative of a reduced efficiency of adaptive strategies, including induction of proteasome biogenesis (so-called *Proteasome stress response*)^16,17^, which might relieve the proteostatic imbalance experienced by these MPM clones. Of note, the stressful condition revealed by basal accumulation of endogenous ubiquitin conjugates is still compatible with apparently normal cell functions, implying regular turnover of key proteasomal substrates (e.g. cyclins). However, this precarious equilibrium can be easily pushed towards a toxic collapse of proteostasis by further compromising the proteolytic route at doses of PIs that are lower than those required to achieve the same result in cells that, like MSTO-211H (and to a lesser extent MMB), are characterized by a more-favorable load-versus-capacity ratio. Importantly, saturation of the proteasomal proteolytic route may stabilize pro-apoptotic effectors, such as the Bcl-2 homology domain 3 (BH3)-only proteins Bim and Noxa and the mitochondrial outer membrane permeabilizers BAX/BAK, or abrogate activity of survival factors like NF-kB, which are all known to play a pivotal role in modulating the sensitivity of MPM cells to bortezomib^44,45,47,49,68^. So far, it has been generally assumed that the load-versus-capacity model may account for the exquisite sensitivity to PIs of MM and other professional secretory cells that are intrinsically predisposed to saturation of the proteasomal degradative route due to their overwhelming rates of protein synthesis. Our data, on the contrary, suggest that a compromised proteostatic equilibrium, arising at least in part from a reduced proteasome pool, might be a typical hallmark of PIs-sensitive cancers in general. Although further studies will be required to fully understand the molecular mechanisms of this proteostatic unbalance in MPM and possibly in other PIs-responsive solid cancers, it is possible that the high levels of genotoxic stress typically experienced by tumor cells, with the consequent enhanced generation of mutated/abnormal polypeptides requiring extremely fast degradation^69^, accompanied by a reduced efficiency of appropriate adaptive strategies counteracting this stressful and potentially harmful condition, might underlie the basal proteotoxic stress seen in our study in MPM clones that are more sensitive to bortezomib.

Our results documenting that an unfavorable load-vs-capacity ratio represents a critical determinant of apoptotic sensitivity and underlies vulnerability to bortezomib not only for the prototypical PIs-responsive hematological cancer multiple myeloma, but also for an extremely recalcitrant solid tumor like MPM, potentially provide a framework for identifying indicators of responsiveness to PIs and for improving their clinical efficacy. Conceivably, our data indicate that assessment of proteasome stress and capacity in MPM patients might represent a potential predictor of individual responsiveness to bortezomib with both prognostic and therapeutic value. Moreover, pharmacological strategies aimed at exacerbating the proteotoxic stress experienced by MPM cells are predicted to lower their intrinsic threshold to apoptosis triggered by PIs and therefore substantially increase their efficacy against this malignancy.

## Methods

### Cell lines

Human malignant pleural mesothelioma (MPM) cell lines were generously provided by Dr. S. Biffo (University of Milan, Milan, Italy) (MM98) and Dr. G. Gaudino (University of Hawaii, Honolulu, HI, USA) (MMB, MSTO-211H, REN). MSTO-211H were maintained in RPMI1640 containing 10% fetal bovine serum (FBS), L-glutamine, and antibiotics; REN and MM98 in DMEM containing 10% FBS, L-glutamine, and antibiotics; MMB in nutrient mixture F-12 Ham supplemented with 110% FBS, L-glutamine, and antibiotics. All MPM cell lines were cultured at 37 °C in an humidified incubator in an atmosphere of 5% CO_2_. Under these growth conditions, the doubling time for all MPM clones was ~24 h. All cell culture reagents were purchased from Sigma-Aldrich (St. Louis, MO, USA).

### Flow-cytometric analyses of apoptosis

Cells were treated with bortezomib (Millennium Pharmaceuticals, Cambridge, MA, USA) as indicated, harvested, stained with FITC-conjugated Annexin V (1 μg/ml) and propidium iodide (2.5 μg/ml) according to the manufacturer’s instructions, and analyzed by FACScalibur (BD Biosciences, Franklin Lakes, NJ, USA) as described^18,19,21^.

### Proteasome activity assays

Proteasome activity was assessed in MPM crude extracts using fluorogenic peptides according to a method that avoids long and laborious chromatographic purification of 26S particles^51^. Accordingly, proteasome-specific peptidase activities were assayed by monitoring the production of 7-amino-4-methylcoumarin (amc) from the following fluorogenic peptides (Bachem, Bubendorf, Switzerland): 100 μM Suc-LLVY-amc (for chymotrypsin-like), 500 μM Bz-VGR-amc (for trypsin-like), and 100 μM Ac-YVAD-amc (for caspase-like activity) in 20 mM Tris-HCl (pH 7.5), 1 mM ATP, 2 mM MgCl_2_, and 0.2% bovine serum albumin (BSA). Two of these substrates (Suc-LLVY-amc and Suc-YVAD-amc) were used at a concentration highly exceeding their K_m_ value for the relative proteasome catalytic β-subunit, and therefore hydrolysis rates were assessed at V_max_. The third substrate (Bz-VGR-amc) has a lower affinity for β2/β2i subunit and was used at a higher concentration to approach its V_max_ of hydrolysis (Not shown). Reactions were started by adding an aliquot of cellular extract, and the fluorescence of released amc (excitation, 380 nm; emission, 460 nm) was monitored continuously at 37 °C with a Carry Eclipse spectrofluorometer (Varian, Palo Alto, CA, USA). Background activity (caused by non-proteasomal degradation) was determined by addition of the PI epoxomicin (Sigma-Aldrich, St. Louis, MO, USA) for chymotrypsin-like activity, MG132 (Calbiochem, San Diego, CA, USA) for caspase-like activity and β-lactone (Enzo Life Sciences, Farmingdale, NY, USA) for trypsin-like activity, each used at a final concentration (2 μM, 10 μM, and 20 μM, respectively) known to completely suppress the relative proteasomal peptidase activity without affecting other proteases with the same cleavage specificity^6^. Assays were calibrated using standard solutions of free fluorophore, and reaction velocities were calculated from the slopes of the initial linear portions of the curves. Substrate consumption at the end of incubation never exceeded 1%.

### Immunoblot analyses

Immunoblot analyses of α and β-subunits of constitutive and immuno proteasomes, α-tubulin, free ubiquitin and polyubiquitinated proteins were performed as previously described^21,54^. Briefly, extracts were resolved by 18% (for free ubiquitin) or 12% (for polyubiquitinated conjugates and all other antigens) SDS-PAGE gel and transferred on Nitocellulose (Sigma-Aldrich, St. Louis, MO, USA, for polyubiquitinated conjugates) or Immobilon®-P (Merck Millipore, Darmstadt, Germany, for all other antigens) transfer membranes. Nitrocellulose membrane was boiled for 5 min to unmask poly-Ub antigens and so make more sensitive and quantitative the immunoblot. The membranes were then incubated in blocking buffer (5% BSA, 0.1%Tween-20 in 1 × PBS), followed by incubation with mAb anti-ubiquitin (P4D1, Santa Cruz Biotechnologies, Santa Cruz, CA, USA), anti α-tubulin (T5168, Sigma-Aldrich, St. Louis, MO, USA), anti-α3, -α4, -α5 (MCP196, MCP79, MCP257, Enzo Life Sciences, Farmingdale, NY, USA), anti-β1, -β2, -β5, -β1i -β2i, -β5i (gift of Prof. S. Ferrone, Harvard Medical School, Boston, MA, USA). Bound antibodies were visualized using the ECL technique and bands were detected with Hyperfilm™ ECL™ (GE Healthcare). Densitometric analysis was performed with a VersaDoc 1000 Imaging System and Quantity One software (Bio-Rad, Hercules, CA, USA).

### Immunofluorescence

MPM cell lines were seeded on poly-L-lysine-coated slides, fixed with 4% paraformaldehyde for 10 min at room temperature (RT), and permeabilized with PBS 0.1% Triton X-100 for 10 min at RT. Cells were then preincubated with PBS-1% normal goat serum (NGS) for 30 min at RT, incubated with monoclonal mouse anti-ubiquitin (clone FK2, 1/100; Enzo Life Sciences, Farmingdale NY, USA) diluted in PBS-1% NGS for 1 hour at RT, rinsed in PBS, and then stained with goat anti-mouse IgG Alexa Fluor 488 (1/200, Life Technologies, Paisley, UK) diluted in PBS-1% NGS for 1 hour at RT. After rinsing in PBS, cells were stained with propidium iodide for 5 min at RT (1/1000; Sigma-Aldrich, St. Louis, MO, USA), washed in PBS, and mounted with Vectashield mounting medium (Vector Laboratories, Burlingame, CA, USA). Images were acquired as single-stack images (capture images) at 40× water immersion with a confocal laser scanning microscope (TCS SP5, Leica Microsystems). Laser parameters were kept unchanged for all the different cell groups so that the detection of staining was maximal while avoiding pixel saturation. Quantitative analysis was performed by an operator unaware of the experimental group. Images were analyzed with ImageJ software (NIH, Bethesda, MD, USA) measuring cell area, mean gray value, and integrated density. More than 100 cells per group were counted and analyzed.

### Statistical analysis

To compare average measurements of peptidase proteasomal activities, western blot densitometries, and immunofluorescence intensities, we adopted a non-parametric Mann-Whitney test, since preliminary Shapiro-Wilk tests have proven that data were not normally distributed. Data were graphically visualized using box plots.

### Data Availability

No datasets were generated or analyzed during the current study.

## Electronic supplementary material

Supplementary information
