# Supplementary material for: Proteasome stress sensitizes malignant pleural mesothelioma cells to bortezomib-induced apoptosis
Source: Sci Rep. 2017 Dec 15;7:17626. doi: 10.1038/s41598-017-17977-9 (PMC5732203; doi:10.1038/s41598-017-17977-9)
Supplement: Supplementary file 1 — Supplementary information [file 41598_2017_17977_MOESM1_ESM.pdf]

Proteasome stress sensitizes malignant pleural mesothelioma cells to bortezomib-induced apoptosis

Fulvia Cerruti[1], Genny Jocollé[2], Chiara Salio[1], Laura Oliva[3], Luca Paglietti[1], Beatrice Alessandria[1], Silvia Mioletti[1], Giovanni Donati[4], Gianmauro Numico[5], Simone Cenci[3], Paolo Cascio[1*]

[1] Department of Veterinary Sciences, University of Turin, Largo P. Braccini 2, 10095, Grugliasco, Turin, Italy. fulvia.cerruti@unito.it; chiara.salio@unito.it; luca.paglietti@edu.unito.it; beatrice.alessandria@edu.unito.it

[2] Medical Oncology Unit, Ospedale U. Parini, Viale Ginevra 3, 11100 Aosta, Italy; gennajiit@yahoo.it.

[3] San Raffaele Scientific Institute, Division of Genetics and Cell Biology, Via Olgettina 60, 20132 Milan, Italy; oliva.laura@hsr.it; cenci.simone@hsr.it

[4] Thoracic Surgery Unit, Ospedale U. Parini, Viale Ginevra 3, 11100 Aosta, Italy; gdonati@ausl.vda.it

[5] Medical Oncology, Azienda Ospedaliera SS Antonio e Biagio e C Arrigo, Via Venezia 16, 15121 Alessandria, Italy; gianmauro.numico@ospedale.al.it

Corresponding author: Paolo Cascio, [1]Department of Veterinary Sciences, University of Turin, Largo P. Braccini 2, 10095, Grugliasco, Torino, Italy.

Tel: +39 0116 709109, Fax: +39 0116 709138, Email: paolo.cascio@unito.it

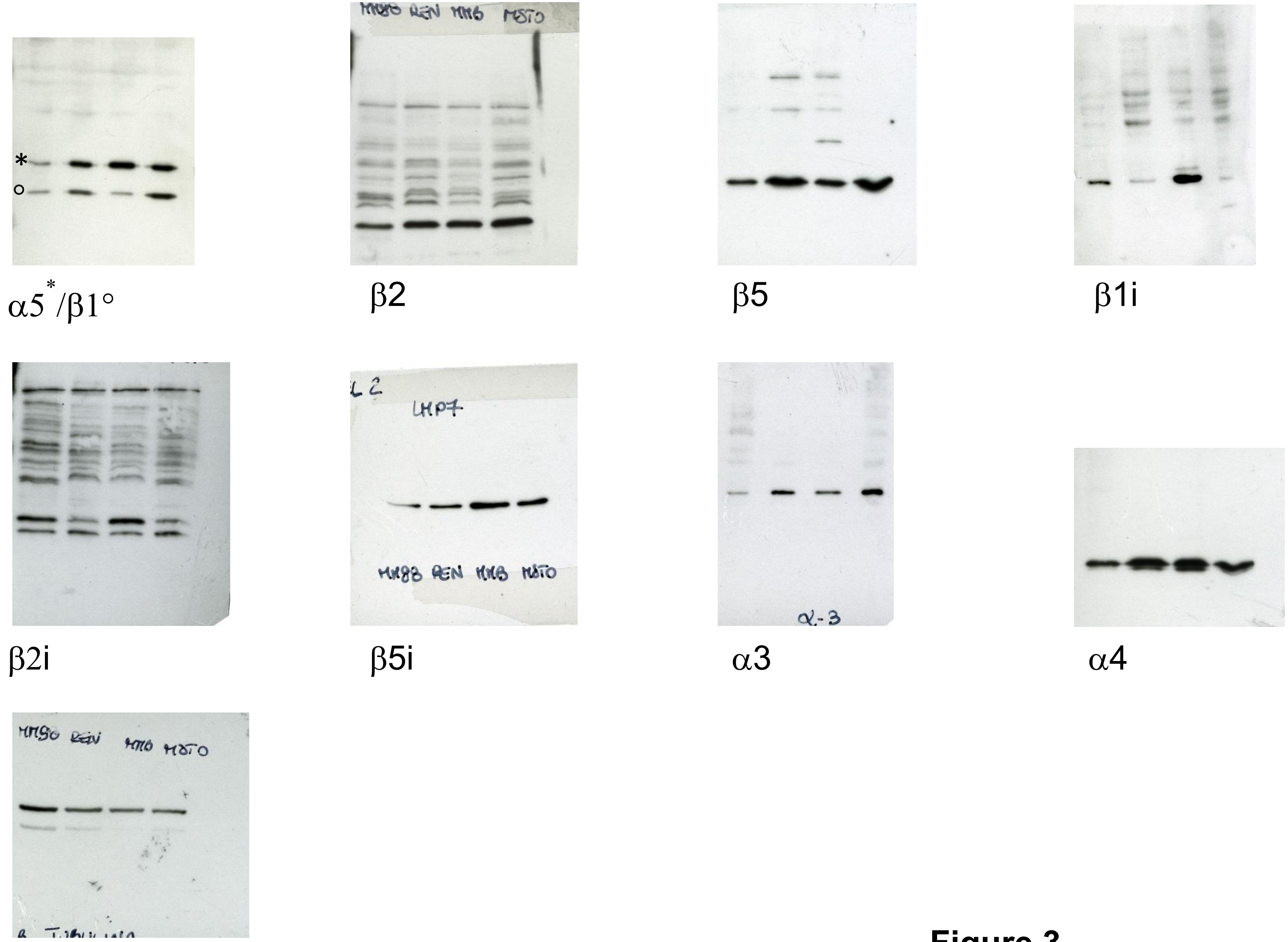

Figure 3

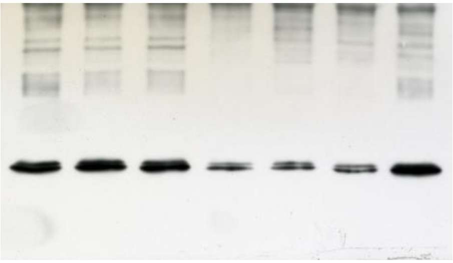

MSTO-211H, REN, MMB

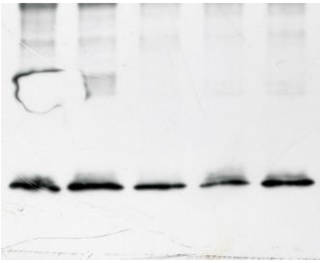

MMB, MM98

Figure 4 A
